# Illuminating biomolecular assemblies in gene regulation

**DOI:** 10.1007/s12551-025-01340-8

**Published:** 2025-08-15

**Authors:** Eve Dixon, Karolina Stanczyk, Yolanda Markaki

**Affiliations:** Department of Molecular and Cell Biology, Institute for Structural and Chemical Biology, University of Leicester, University Road, Leicester, LE1 7RH UK

**Keywords:** Biomolecular assemblies, Condensates, Gene regulation, NcRNAs, Genome compartmentalization, Supramolecular complexes

## Abstract

The nucleus is a highly compartmentalized organelle and this spatial organization reflects gene-regulatory environments. Chromatin exists in two distinct forms: transcriptionally active, euchromatin and silenced, compacted heterochromatin. The spatial organization of chromatin along with its transcriptional activity is governed by biomolecular assemblies (BAs). Gene regulatory assemblies form and operate through highly dynamic protein–protein and protein-DNA interactions often established via their recruitment by non-coding RNAs. The formation of BAs is essential for retaining diffusible regulatory proteins at specific genomic regions, enabling local confinement and precise gene regulation. Phase separation, particularly in the form of liquid–liquid condensation, is suggested to play a crucial role in transcriptional regulation, serving as a key driver of biomolecular assembly formation. However, some studies indicate that phase separation may also be a non-essential byproduct of the crowded nuclear environment or may not be involved in certain BAs. Despite extensive investigations into these macromolecular crowding phenomena, the precise mechanisms underlying both the formation of gene-regulatory BAs and how these localized protein concentrations function to regulate chromatin structure and gene expression remain unclear. This review highlights progress made in elucidating the mechanisms of chromatin-modifying BAs, highlighting how super-resolution microscopy and single-molecule technologies are proving essential for probing these nuclear structures in situ, within their native cellular context.

## Introduction

The nucleus is a membraneless organelle where DNA, RNA, and proteins come together to organize intricate chromatin compartments of specialized activities. Chromatin broadly exists in two structurally and functionally distinct forms: euchromatin, which is relaxed and transcriptionally active, and heterochromatin, which is condensed and transcriptionally silent. The formation and maintenance of these compartments are controlled through a hierarchal network of epigenetic mechanisms, including post-translational modifications of histones, DNA methylation, RNA-mediated processes, such as transcription or targeting through non-coding RNAs (ncRNAs), and physical scaffolding, which all work together to fine-tune gene expression and chromatin organization (Paldi and Cavalli 2025).

**Fig. 1:**
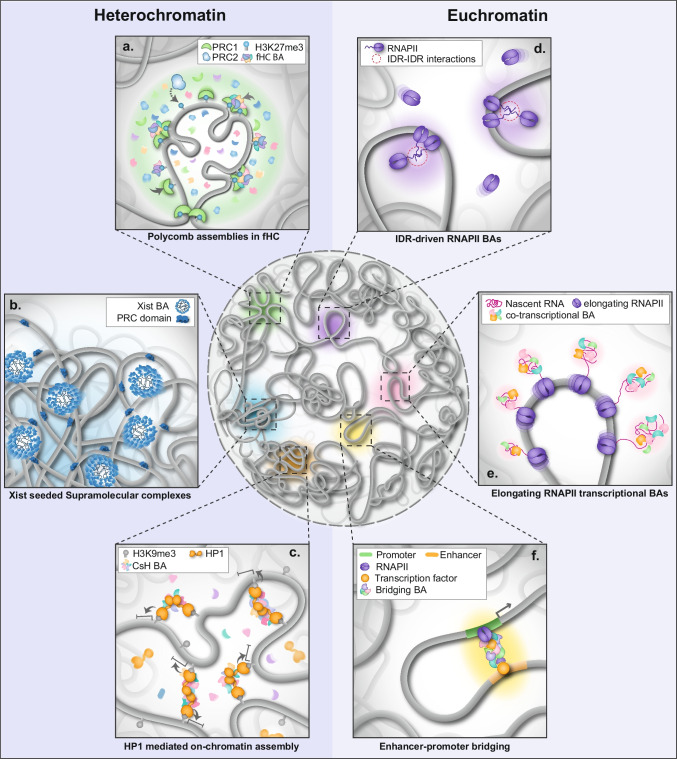
Examples of gene-regulatory biomolecular assemblies. **a** Polycomb assemblies within facultative heterochromatin compartments deposit repressive epigenetic marks, including H3K27me3, which act as binding sites to prime protein-DNA and protein–protein interactions (PPIs) that enhance the assembly of fHC BAs onto chromatin. H3K27me3 modifications also act to further recruit additional Polycomb proteins in a positive feedback loop which can promote the spread of fHC (Tatavosian et al. 2019). **b** Xist RNA acts as a scaffold to recruit effector proteins, leading to the nucleation of SMACs, Xist BAs on the inactive X territory, which are further enhanced by IDR-IDR interactions and PPIs (Markaki et al. 2021). A sub-population of SMAC proteins, such as Polycomb complexes form secondary hubs to induce spreading of heterochromatin. **c** HP1 dimers can stably bind to H3K9me3 nucleosomes and facilitate the on-chromatin assembly of CsH BAs that will initiate gene repression and the coalescence of chromatin fibers (Hiragami-Hamada et al. 2016; Chen et al. 2024). **d** RNAPII clusters of few molecules dynamically assemble onto decondensed chromatin (Cho et al. 2016; Stein et al. 2025) organized through IDR-IDR interactions between RNAPIIs CTDs (Boehning et al. 2018). **e** Nascent RNA molecules being transcribed by elongating RNAPII molecules assemble into nanodomains that recruit the RNA processing machinery including splicing factors to form co-transcriptional nRNPs (Castells-Garcia et al. 2022). Increased accumulation of these nRNPs enhances chromatin stiffness and promotes protrusion of transcriptional loops into the interchromatin space (Leidescher et al. 2022). **f** Enhancers and promoters are bridged by BAs that multivalently interact with specific interactors of both elements, triggering chromatin looping and the coalescence of transcriptional regulators (Boija et al. 2018; Benabdallah et al. 2019). BA: biomolecular assemblies, CTD: C-terminal domain, CsH: constitutive heterochromatin, fHC: facultative heterochromatin, HP1: heterochromatin protein 1, IDR: intrinsically disordered region, nRNP: nascent ribonucleoprotein, RNAPII: RNA polymerase II, PRC1: polycomb repressive complex, SMAC: supramolecular complex, TF: transcription factor

This regulation is achieved by an array of diverse effector proteins and RNAs that interact to form biomolecular assemblies (BAs) with representative examples illustrated in Fig. 1. Formation and chromatin engagement of BAs lead to the colocalization of otherwise diffusible and inoperative regulatory proteins to facilitate their rapid and coordinated action and ensure the specific temporal control of nuclear processes (Cremer et al. 2015).

While established stoichiometric complexes that can be purified and studied in solution form the fundamental backbones of BAs, these large-scale assemblies emerge from the collective action of weak, transient, multivalent, and dynamic interactions between numerous proteins (Darzacq and Tjian 2022). The process of BA formation is driven by macromolecular crowding, where various proteins and other macromolecules are locally concentrated at genomic sites. This localization facilitates specific and enhanced protein–protein interactions (PPIs), allowing for the fine-tuning of regulatory outputs. For instance, the binding of BAs to chromatin often depends on initial protein-DNA interactions and crowding events that set the stage for subsequent PPIs. Understanding how these crowding events are precisely orchestrated in time and space within the complex cellular environment remains a significant challenge.

One mechanism by which macromolecular crowding promotes BA formation is through phase transitions, where typically increased protein concentration triggers phase separation (PS) and condensation, often via liquid–liquid phase separation (LLPS), driven by multivalent, low-affinity, non-covalent PPIs (Hyman et al. 2014; Li et al. 2025; Pei et al. 2025; Banani et al. 2017). LLPS was originally characterized in vitro by observing certain proteins forming “liquid-like” droplets that exclude diffusible proteins under specific conditions. Subsequent in vivo observations, such as droplet fusion and fission, globular morphology, concentration buffering, internal mixing, and selective permeability, have made LLPS a compelling model for the self-organization of subcellular membraneless assemblies (Mittag and Pappu 2022).

However, growing evidence suggests that the significance of PS in chromatin organization and nuclear BAs formation has been overgeneralized, largely based on the realization that in vitro droplet assays cannot fully recapitulate the highly dynamic, multivalent milieu of living cells, raising questions about the universal applicability of LLPS mechanisms to all nuclear BAs (Rippe 2022; Riback et al. 2020). Recent super-resolution single-molecule tracking studies and kinetic modeling suggest that direct, specific, and dynamically regulated tethering of limited numbers of proteins to DNA may be sufficient for functional assembly, without requiring the formation of highly concentrated, biophysically distinct phases as seen in LLPS (Muzzopappa and Erdel 2025; McSwiggen et al. 2019; Darzacq and Tjian 2022). We discuss several examples of these DNA-tethered BAs throughout this review.

Formation of BAs is known to be facilitated by intrinsically disordered regions (IDRs) (Guo et al. 2021; Borcherds et al. 2021). IDRs are unstructured, low-complexity protein segments capable of forming weak, multivalent interactions with RNA, ordered proteins, and other IDRs (Ferrie et al. 2022; Borcherds et al. 2021). Their flexible and dynamic nature allows IDRs to adapt conformationally, enhancing their binding capabilities (van der Lee et al. 2014; Ferrie et al. 2022). Commonly found in chromatin regulators and transcription factors (TFs), IDRs facilitate transient interactions that enable these factors to bind effectively but also dynamically, as well super-stoichiometrically (Lee et al. 2024).

Key players in the biogenesis of BAs are RNAs, particularly non-coding RNAs (ncRNAs). These molecules have a unique ability to serve as scaffolding platforms for regulatory proteins, thereby enhancing their local concentrations and specificity to genomic regions (Muzzopappa and Erdel 2025). RNA-seeded assemblies play a crucial role in various nuclear processes including epigenetic regulation (such as X chromosome inactivation), higher-order chromatin organization, signaling, RNA processing, and splicing (Quinodoz et al. 2021; Mattick et al. 2023). They are essential for stabilizing BAs, acting as binding sites for multiple interacting proteins and promoting novel PPIs. LLPS and other forms of PS are increasingly recognized as mechanisms by which many ncRNAs may contribute to nuclear compartmentalization and have been reviewed elsewhere (Frank and Rippe 2020; Unfried and Ulitsky 2022; Rippe 2022).

Another significant challenge is identifying appropriate cellular systems for observing gene-regulatory BAs, which often function in highly specific developmental and tissue contexts. Many studies rely on terminally differentiated or cancer cell lines that may not capture the true physiological roles of these assemblies. Here, we explore various examples to illustrate the importance of studying BAs in functionally relevant systems. For example, tissue-specific expression of certain genes gives rise to giant transcriptional loops which cannot be observed in cell cultures (Leidescher et al. 2022), and heterochromatin formed during embryogenesis is maintained as epigenetic memory in differentiated cells, with distinct factors and mechanisms for initiation and maintenance (Chu et al. 2015). In X chromosome inactivation, specific proteins, such as the major transcriptional co-repressor and RNA binding protein, SPEN, are essential for silencing during initiation but dispensable during maintenance (Dossin et al. 2020). We further discuss how BA composition, protein concentration, and dynamics vary between these phases.

Over the past decade, genomics technologies have provided unprecedented resolution in mapping chromatin organization, DNA, RNA, and effector protein distributions reaching down to scales of just a few dozen base pairs. Parallel, structural, biophysical, and biochemical approaches have shed light on how proteins accumulate on DNA to assemble regulatory machinery. Despite these innovations, many techniques still struggle to illuminate the three-dimensional architecture and dynamic interactions between BAs and DNA that underlie gene regulation and chromatin structure. To bridge this gap, in situ studies, especially those preserving physiological protein expression, have increasingly relied on advanced microscopy. Fluorescence microscopy allows simultaneous visualization of multiple components and real-time observation of molecular interactions in living cells. The development of super-resolution techniques such as three-dimensional structured illumination microscopy (3D-SIM), single-molecule localization microscopy (SMLM), and stimulated emission depletion (STED) nanoscopy, particularly when combined with live-cell imaging, has dramatically improved the resolution and analysis of nuclear dynamics down to the single-molecule level, making these methods essential for investigating BAs, which are often diffraction-limited.

Building on these imaging advances, we explore the processes by which gene-regulatory BAs form, are spatially organized and exert their functions within chromatin. We discuss the critical roles of protein–protein interactions (PPIs) and protein concentrations in controlling BA-mediated gene expression and genomic compartmentalization, highlighting that functional effects may stem from fewer molecules than previously estimated. Additionally, we examine how noncoding RNAs, using Xist RNA as an example, orchestrate large-scale gene silencing by promoting BA formation and macromolecular crowding, and we propose potential mechanisms underlying these processes.

### Euchromatin

**Fig. 2:**
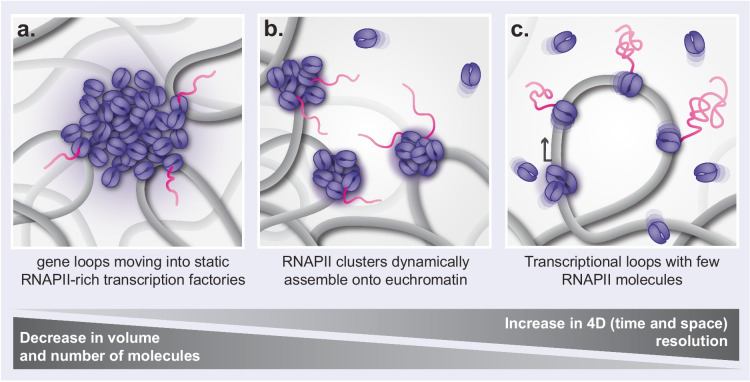
Models of RNAPII assemblies with increasing temporal and spatial resolution. **a** Active genes relocate into pre-assembled diffraction limited transcription factories that are enriched with RNAPII molecules (Jackson et al. 1993). **b** RNAPII clusters comprised of up to 80 polymerases assemble onto active genes within the perichromatin region (Cho et al. 2016b). RNAPII molecules assemble dynamically on chromatin, with clusters displaying lifetimes of ~ 6 s when measured by time-correlated PALM (Cho et al. 2016a, b). **c** Small RNAPII clusters containing 1–4 molecules of RNAPII assemble at chromatin loops (Stein et al. 2025). Once released from the cluster, facilitated by chemical modifications to RNAPII that disrupt CTD-CTD interactions (Feaver et al. 1994), elongating RNAPII molecules translocate along the gene body. Individual elongating RNAPIIs are interspaced by a few hundred nucleotides (Tantale et al. 2016). Nascent RNA produced from transcription is organized into 600 nm^2^ nanodomains (Castells-Garcia et al. 2022). Nascent ribonucleoprotein (nRNP) assemblies form as splicing machinery is co-transcriptionally recruited to nascent RNA (Leidescher et al. 2022), leading to the stiffening of chromatin and formation of protruding transcriptional loops (TLs).

Transcription is regulated through BAs of RNA polymerase II (RNAPII) with TFs and elongation factors. A seminal study using in situ fluorescence labelling of nascent RNA through the incorporation of the synthetic nucleotides introduced the concept of “transcription factories” (Jackson et al. 1993). Transcription factories are spatially confined hubs rich in RNAPII and nascent RNA transcripts (Fig. 2a) (Iborra et al. 1996; Osborne et al. 2004). High-resolution confocal microscopy studies later revealed that splicing factors-enriched “speckles” are adjacent to these factories and serve as splicing sites (Pombo and Cook 1996). These findings led to the hypothesis that transcription is confined within RNAPII-rich factories, where genes actively relocate into these static hubs, allowing the RNA to be spliced by neighboring speckles following transcription (Fraser and Bickmore 2007).

Further examination using higher resolving techniques, including 3D-SIM, identified clusters of RNAPII localized to chromatin fibers found in the perichromatin region (Markaki et al. 2010), a ~ 200-nm thick layer that lies at the interphase between chromatin domains and the DNA-free interchromatin (IC) space (Fakan and van Driel 2007; Cremer et al. 2015; Cremer and Cremer 2001). Perichromatin is enriched with factors that interact with and process RNA such as heterogeneous nuclear ribonucleoprotein C 1/2 (hnRPNC1/2), actively transcribing RNAPII, as well as epigenetic hallmarks of active transcription (Miron et al. 2020). SMLM techniques including photoactivated localization microscopy (PALM) and stochastic optical reconstruction microscopy (STORM) provided unprecedented view into the organization of transcriptional BAs. RNAPII clusters were estimated to contain up to 80 molecules while, single-molecule tracking (SMT) experiments, particularly time-correlated PALM, provided critical insights into their highly dynamic nature, revealing that they rapidly form and disassemble with lifetimes of only 5–8 s (Elf et al. 2007; Gebhardt et al. 2013; Andrecka et al. 2008; Cho et al. 2016a, b). These findings led to a revised model where diffusible transcriptional and splicing machinery dynamically assembles onto exposed chromatin (Fig. 2b), rather than existing as large, pre-defined static factories.

More recently, TIRF microscopy combined with DNA-PAINT on cryosections has shown that functional RNAPII clusters contain only 1–4 molecules (Stein et al. 2025) (Fig. 2c). STORM of RNA molecules further revealed that nascent transcripts are organized into nanostructures of around 600 nm^2^, termed RNA nanodomains, which were found to be closely associated with elongating RNAPII (Castells-Garcia et al. 2022). An average of 2–3 RNAs were detected per nanodomain, with a significant number of these domains found to be linked to individual genes. These nanodomains exhibit a maximum density peak at a distance of 20–50 nm from the RNAPII clusters, suggesting an intricate spatial relationship that indicates how active transcription may influence chromatin organization at the nanoscale level.

Processing of nascent RNA also requires the coordinated formation and function of splicing BAs. Actively transcribed genes are situated near nuclear speckles, which are dynamic structures rich in splicing factors that enhance co-transcriptional processing (Spector and Lamond 2011, Misteli 2020). This spatial organization increases the concentration of splicing factors adjacent to nascent pre-mRNAs, resulting in more efficient splicing kinetics, illustrating how spatial arrangement directly influences processing rates.

Leidescher and coworkers identified that highly expressed long genes form transcription loops (TLs) which can expand far beyond the genic regions from where they emanate (Leidescher et al. 2022). Multiple RNAPII molecules move along each loop carrying nascent RNAs which are being co-transcriptionally spliced, forming complexes of nascent ribonucleoproteins (nRNPs) (Fig. 1e). The accumulation of nRNPs along the gene body stiffens chromatin, and in the case of larger genes, potentially promotes protrusion of the loops outside of the chromosome territory. It is therefore possible that these TLs have no functional significance but are simply an artefact of transcription (Solovei and Mirny 2024). The protrusion of TLs is consistent with the observation that long neuronal genes in brain cells can “melt” upon high expression (Winick-Ng et al. 2021). “Melting” refers to the decondensation of these genes captured using Genome Architecture Mapping (GAM) on thin tissue cryosections.

Using PALM imaging and biophysical methods, RNAPII clusters have been shown to form through LLPS arising from IDR-IDR interactions between the disordered C-terminal domain (CTD) tails of RNAPII, which may stabilize cluster assembly to promote transcription (Fig. 1d) (Boehning et al. 2018). Formation of transcriptional BAs through LLPS enhances interactions by creating a high local concentration of TFs. This increased crowding facilitates a higher kinetic binding rate, promoting more efficient assembly and interaction among the regulatory proteins involved in transcription (Rippe, 2022; Yang and Hansen 2024; Sabari et al. 2018). An additional level of regulation is provided by post-translational modifications of the CTD tails. These diverse modifications play crucial roles at various stages of the transcriptional cycle by enabling specific interactions between the modified residues and components of the transcriptional machinery. Each modification can signal distinct functions, facilitating processes such as transcription initiation, elongation, and RNA processing, thereby fine-tuning gene expression in response to specific cellular contexts (Buratowski 2003). For instance, phosphorylation of serine 5 on the RNAPII CTD is a hallmark of transcriptional elongation. This modification not only marks the transition into the elongation phase but also inhibits interactions with other CTDs. As a result, the phosphorylated RNAPII can detach from the transcriptional cluster, facilitating its entry into the gene body and allowing for efficient elongation of the RNA transcript (Feaver et al. 1994).

Mediator complexes additionally promote the initiation of transcription by establishing “bridging” BAs that concentrate RNAPII molecules and the transcriptional machinery (Boija et al. 2018; Dixon et al. 2016). These “bridges” bring promoters and enhancers in close spatial proximity (Fig. 1f). The promoter-enhancer distance was found to increase upon transcriptional activation during neural differentiation, a phenomenon proposed to result from the crowding of the transcriptional machinery into large BAs (Benabdallah et al. 2019). Recent studies have shown that these promoter-enhancer bridges are highly dynamic, with interactions lasting from few milliseconds to seconds, consisting of mobile factors that diffuse across the gap and establish transient interactions, rather than forming static structures (el Khattabi et al. 2019; Karr et al. 2022).

Interestingly, the formation of condensates appears to be an important mechanism of a transcriptional feedback loop. RNA carries a negative charge, and its production affects local electrostatic interactions (Banerjee et al. 2017). Following transcription initiation, when a certain threshold of RNA is reached, the local charges are neutralized. This change in charge alters the electrostatic interactions, promoting the coacervation and spatial partitioning of regulatory RNAs, transcription factors, the Mediator complex and RNAPII into a condensate (Henninger et al. 2021; Overbeek and Voorn 1957). As transcription progresses, the accumulation of negatively charged nascent RNA further shifts the charge balance within the condensate. Using the example of the Mediator MED1-IDR condensate, Henninger and co-workers demonstrated that this non-equilibrium PS can lead to the dissolution of the condensate, acting as a feedback control mechanism that slows or terminates transcription (Henninger et al. 2021).

By employing SRM and SMT techniques, we have made significant progress in understanding the formation and mechanisms of transcriptional BAs. Unlike early models that proposed large, static assemblies, we are revealing the highly dynamic nature of RNAPII clusters binding to chromatin. This dynamic assembly can occur through just a few bound molecules, stabilized by multivalent, high-affinity IDR-IDR interactions.

## The silenced genome

### Constitutive heterochromatin (CsH)

Broadly defined as regions of chromatin that are maintained in a condensed and transcriptionally silent state throughout a cell’s lifetime, constitutive heterochromatin is found predominantly in gene-poor and repeat-rich areas of the genome such as telomeres, centromeric regions and transposable elements. Hallmarks of these regions include repressive epigenetic modifications, such as H3K9me3 and DNA methylation (Lehnertz et al. 2003), as well as BAs of silencing proteins including heterochromatin protein 1 (HP1) (Bannister et al. 2001; Lachner et al. 2001), Suv39h1/2 (Lehnertz et al. 2003), MECP2 (Lewis et al. 1992; Brero et al. 2005), and ATRX (McDowell et al. 1999; Marano et al. 2019).

One of the best characterized examples of constitutive heterochromatin is mouse pericentric heterochromatin (PCH), pericentromeric satellite repeats which cluster into densely condensed structures known as “chromocenters” that are readily visualized using DNA dyes (Abercrombie and Stephenson, 1969). Consequently, murine chromocenters have become a popular model for studying mammalian constitutive heterochromatin.

The action of HP1 at chromocenters has been attributed to its roles in structural organization and transcriptional repression (Grewal 2023, Schoelz and Riddle 2022) and in recent years it has been proposed to enact these functions through its ability to induce a phase transition (Rippe, 2022). Droplet assays revealed that at high concentrations HP1 can undergo LLPS in the presence of DNA or chromatin (Wang et al. 2019; Keenen et al. 2021; Larson et al. 2017) which in part may be enhanced through IDR-IDR interactions (Phan et al. 2024). Fluorescence recovery after photobleaching (FRAP) experiments coupled to kinetic modeling concurrently showed that chromocenters within Drosophila embryos can exclude inert proteins and fuse to form larger structures, consistent with a LLPS model, reinforcing in vitro findings (Strom et al. 2017). Together, these studies established a widely accepted model in which the function of HP1 at chromocenters relies on high local concentrations. Once this threshold is reached, HP1 interacts with PCH to form a liquid phase with distinct biochemical and biophysical properties compared to the surrounding nucleoplasm, a hallmark of LLPS. This PS is crucial for maintaining PCH in a compacted and transcriptionally silent state.

However, studies examining chromocenters at higher resolution and live-cell imaging techniques have yielded conflicting evidence regarding the role of HP1 and its mechanism of action at PCH. Crucially, endogenous HP1 levels measured by STED microscopy are estimated to be over tenfold lower than the half-saturation point required for droplet formation in vitro (Erdel et al. 2020). While this could potentially be reconciled with findings that increasing heterotypic interactions enables phase transitions at lower individual protein concentrations (Riback et al. 2020), in situ observations demonstrate that HP1 does not form stable droplets in vivo (Erdel et al. 2020). Instead, STED nanoscopy has further revealed HP1 clusters within chromocenters, which align with either chromatin ultrastructure or the DNA-free interchromatin space (Weinmann et al. 2024). These distinct distributions reflect different HP1 populations: one that is immobile and chromatin-bound, and another that is mobile and freely diffusing within the interchromatin space, as previously observed through FRAP and kinetic modeling (Erdel et al. 2020; Strom et al. 2017; Chen et al. 2024). Post-translational modifications of HP1 may regulate these populations by modulating the protein’s chromatin binding affinity (Larson et al. 2017). Supporting this, genetic perturbations combined with super-resolution single-molecule imaging in yeast demonstrated that immobilization of HP1 upon H3K9me3 nucleosome binding promotes the formation of ternary complexes through an “on-chromatin assembly” mechanism, whereas formation of HP1 BAs is unfavorable when the protein is not chromatin-bound (Fig. 1c) (Chen et al. 2024). Although these experiments have yet to be replicated in a mammalian system, this suggests that formation of HP1-mediated BAs is primarily governed by interactions with the local chromatin environment, rather than by an innate tendency of HP1 to undergo concentration-dependent droplet formation. Chromatin-bound HP1 dimers have also been shown to bridge H3K9me3 nucleosomes, facilitating the coalescence of chromatin fibers into a “gel-like polymer” (Machida et al. 2018; Hiragami-Hamada et al. 2016; Singh and Newman 2022), a process that may contribute more to the mechanical stiffness of chromatin than to its compaction (Strom et al. 2021).

The role and properties of HP1 appear to differ between initiation and maintenance stages of heterochromatin formation (Seman et al. 2023). This could be due to differences in the pool of cofactors or modifications of HP1 and/or chromatin. For instance, chromocenters in naïve mouse embryonic stem cells exhibit a dynamic state characterized by relaxed chromatin, a higher mobile fraction of HP1, and increased transcription of non-coding repeats into ncRNAs, although the implications of these features on HP1 function remain unclear (Brero et al. 2005; Kobayakawa et al. 2007; Novo et al. 2022; Probst et al. 2010). Live-cell high-resolution confocal imaging has shown that upon forced activation of chromocenters by TFs, these structures can form de-compacted configurations resembling collapsed polymer globules, a process that appears to be largely independent of HP1 or H3K9me3 occupancy (Erdel et al. 2020; Weinmann 2024). Likewise, knockout of the H3K9 methylases Suv39h1/2 results in the loss of HP1 enrichment at PCH without significantly disrupting chromatin compaction or other heterochromatic hallmarks, such as DNA methylation or MECP2 recruitment (Pantier et al. 2024). Similarly, rapid degradation of HP1-AID in human U2OS cells does not significantly alter local heterochromatin compaction or global transcription (Strom et al. 2021). These findings align with earlier studies demonstrating that HP1 is largely dispensable for heterochromatin maintenance (Karimi et al. 2011). In contrast, HP1 seems to play a more defined role in the initiation of heterochromatin formation or direct gene silencing. For example, targeted tethering of HP1 to promoters is sufficient to induce transcriptional silencing and chromatin compaction (Li et al. 2003; Verschure et al. 2005), an effect achieved without the formation of HP1 assemblies or droplets (Erdel et al. 2020). Together, these data demonstrate that HP1, at least in some genomic contexts, can carry out its mechanism of action through a limited number of DNA-bound molecules without relying on highly concentrated assemblies. It is possible that LLPS contributes to the function of HP1 at PCH, but this mechanism is restricted to specific developmental stages or tissues.

### Facultative heterochromatin (fHC)

Facultative heterochromatin consists of transcriptionally competent genomic regions that become silenced in response to stimuli, such as developmental cues (Zylicz and Heard 2020; Kim and Kingston 2022). Typically, regulation of fHC is achieved through Polycomb repressive complexes (PRCs) which deposit repressive histone marks, including trimethylation of lysine 27 of histone H3 (H3K27me3) and ubiquitylation of H2A lysine 119 (H2Aub119) (Muller et al. 2002; Wang et al. 2004). These marks function to recruit an array of additional effector proteins that then initiate and propagate chromatin compaction, leading to the spread of fHC (Fig. 1a) (Ingersoll et al. 2024; Kraft et al. 2022; Veronezi and Ramachandran 2024).

X chromosome inactivation exemplifies fHC formation, arising as a developmental mechanism in mammalian XX females to balance gene dosage with XY males (Lyon 1961). The inactive X (Xi) forms a distinct heterochromatin structure called the Barr body (Barr and Bertram 1949) with gene repression linked to chromosome-wide compaction, making it an ideal model to study large-scale fHC initiation and maintenance. X-inactivation is a ncRNA-driven silencing mechanism in which chromosome-wide inactivation is mediated in *cis* by the long ncRNA, Xist (Brockdorff et al. 1992). Xist was first visualized by Fluorescence In Situ Hybridization (FISH) as a diffraction-limited “cloud” coating the inactive X chromosome, that acts as a scaffold for transcriptional repressors (Brown et al. 1992; Clemson et al. 1996). Indeed, subsequent biochemical and in-situ imaging analyses identified over 100 interacting proteins and associated histone marks such as PRC2-mediated H3K27me3, that also cover the Xi, (McHugh et al. 2015; Chu et al. 2015). In the past decade, SRM studies of the inactive X compartment pioneered by the Cremer lab identified a limited amount of Xist RNA, which explains its specificity to the chromosome (Markaki et al. 2012; Markaki et al. 2013; Smeets et al. 2014; Sunwoo et al. 2015; Cerase 2016; Rodermund et al. 2021). Using 3D-structured Illumination microscopy, only 50 Xist foci, each containing approximately 2 RNA molecules, were found to form the previously convoluted Xist cloud (Markaki et al. 2021). This raised the question of how a limited amount of Xist can mediate chromosome-wide silencing.

A key finding derived through 4D (space and time) SIM of Xist RNA followed by single-particle tracking, was that Xist foci exhibit highly confined motion, with typical displacements less than 200 nm over several minutes (Markaki et al. 2021). These data demonstrated for the first time, that Xist clusters do not freely diffuse across the chromosome but instead, they are stably bound to chromatin and monitored to remain stably associated with the same low density chromatin regions. This “wiggling” motion of Xist closely parallels the sub-diffusive motion of chromatin loci, which exhibit a similar mean squared displacement (MSD) of roughly 200 nm (Nozaki et al. 2017; Tortora et al. 2020; Chen et al. 2013). Integrating multispectral SIM and FRAP experiments combined with kinetic modeling, Markaki and coworkers further revealed that Xist serves as a scaffold for the dynamic assembly of effector proteins, forming a supramolecular complex (SMAC) (Fig. 1b). These findings showed that SMACs are dynamic BAs rather than a static complex which persist from initiation to maintenance of X-inactivation.

The super-stoichiometric binding of proteins to Xist is driven by IDRs, as demonstrated by quantitative 3D-SIM measuring the spatial concentrations of SPEN in SMACs in WT and IDR-deleted mutants (Markaki et al. 2021). This hypothesis was further explored through conventional imaging of the cloud formation of synthetically induced Xist transcripts in relation to SPEN coverage over the Xi. Interestingly, a SPEN mutant protein with IDRs replaced by those of the highly unstructured protein FUS retained its accumulation on the Xi (Jachowicz et al. 2022). However, FUS interacts with Xist across much of its length (Smola et al. 2016) and thus, it still remains unclear how IDRs contribute to these interactions or if other factors are involved. Recent biophysical work revealed that Xist and HNRNPK form a liquid–liquid phase that separates the Xi, facilitating the accumulation of effector proteins and driving chromosome-wide silencing (Ding et al. 2025). Other key components of the Xi, such as PRCs, are involved in self-assembly and PPIs, including LLPS, and could be seeded by Xist and then serve as secondary hubs to spread silencing (Fig. 1b) (Eeftens et al. 2021; Tatavosian et al. 2019; Cerase et al. 2014).

But how do 50 locally-confined SMACs exert their function across an entire chromosome?

**Fig. 3:**
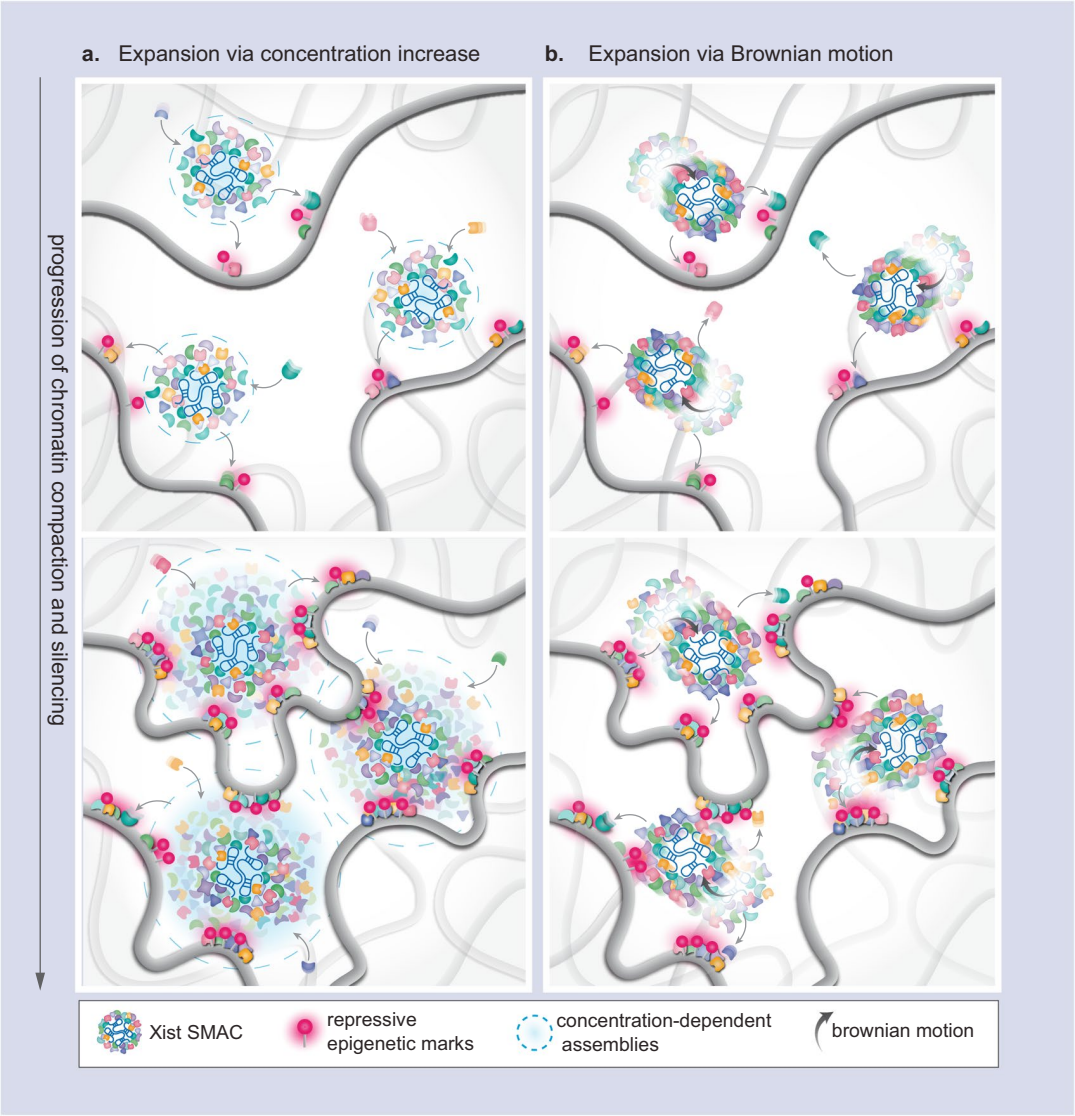
Models of SMAC action**.** Xist exploits its three-dimensional space to exert its silencing role by probing targets in its spatial vicinity (Engreitz et al. 2013; Markaki et al. 2021). Xist acts through the nucleation of SMACs, super-stoichiometric assemblies regulated through IDR-IDR interactions. **a** An increase in the protein concentration and volume of SMACs will lead to a widened silencing radius, enhancing genomic target reach (Cerase et al. 2019) **b** SMACs exploit the Brownian motion of associated chromatin to widen their silencing radius, with only a moderate increase in local protein concentration (Markaki et al. 2021). In both models, which are not mutually exclusive, progressive chromatin compaction draws distal regions within the 3D silencing radius of SMACs, ultimately leading to the propagation of compaction and repressive epigenetic marks throughout the entire compartment. SMACs: supramolecular complexes

RNAPII molecules were found to be excluded from the Xi in fixed cells (Chaumeil et al. 2006; Clemson et al. 1996), and thus, the contribution of PS in this partitioning has been the subject of intense investigation. As SRM fixed-cell experiments revealed that Xist foci do not cover the entire chromosome, it has been proposed that the Xi becomes a large condensate via PS which would allow the RNA to recruit proteins super-stoichiometrically and expand its silencing radius (Cerase et al. 2019) (Fig. 3a). However, SMT experiments combined with fluorescence correlation spectroscopy showed that RNAPII diffuses freely in and through the Xi with no apparent change in mobility or trajectory, but there is depletion of its stably bound fraction which represents 40% of its population in the nucleoplasm (Collombet et al. 2023). Furthermore, this same loss of the immobile fraction can be artificially recreated in the nucleoplasm by chemically inhibiting transcription, suggesting loss of RNAPII binding from the Xi territory is due to active transcriptional repression rather than a biophysical “barrier” preventing diffusion. An alternative explanation for how sparse SMACs achieve chromosome-wide silencing is that DNA-tethered SMACs may regulate gene silencing across broad chromosomal regions through constrained “wiggling” facilitated by Brownian motion (Fig. 3b). SMACs are typically spaced about 200 nm apart, a distance that matches the range of their local confinement (Markaki et al. 2021). This “wiggling” motion allows SMACs to efficiently probe their surrounding chromatin territory without requiring high local concentrations of proteins, as would be necessary for models based on concentration-dependent mechanisms (Fig. 3b) (Cerase et al. 2019; Jachowicz et al. 2022; Quinodoz et al. 2021). These two models are not mutually exclusive; both mechanisms could operate simultaneously or could be differentially employed across developmental contexts. Distinguishing between these possibilities requires advancements in multispectral, quantitative live-cell and single-molecule imaging techniques, as traditional fixed-cell and conventional imaging approaches lack the necessary temporal, spatial and multi-component information.

## Conclusions and future perspectives

It is increasingly evident that in situ, multicomponent observations are essential for unraveling the mechanisms of complex and dynamic gene-regulatory BAs, such as Xist SMACs or RNAPII condensates. Findings from fixed-cell experiments must be interpreted with caution, as prolonged cross-linking can artifactually elevate concentrations and generate artificial structures (Dubochet and Sartori Blanc 2001). For example, fixation protocols that fail to preserve nuclear ultrastructure cause artificial redistribution of RNAPII clusters (Guillot et al. 2004). These artifacts underscore the necessity of integrated, live-cell approaches combined with biophysical techniques.

Future advancements in live-cell SRM techniques, particularly those employing multispectral imaging that will allow the study of multiple components of BAs simultaneously in real time, are poised to provide unprecedented insight into the molecular organization, composition and dynamics of these genome regulators. Such technologies will be instrumental in clarifying whether and how effector protein concentrations and dynamics influence regulatory outcomes, questions that remain largely unsettled within the physiological context. To this end, functional perturbation of endogenous genes and their products will be indispensable for dissecting the causal roles of BAs.

The emerging diversity of models, ranging from phase-separated condensates to dynamic, tethered protein assemblies, offers alternative but not mutually exclusive mechanisms underlying BA function. Discriminating between these scenarios remains challenging with diffraction-limited microscopy, which cannot resolve the native nanometer scale of BAs. Overexpression of proteins further complicates interpretation. These limitations highlight the value of subdiffraction imaging techniques in living cells, such as SMT of endogenously fluorescently tagged proteins. For instance, structures appearing as fused droplets under conventional imaging have often proved to be closely packed, distinct assemblies when viewed with higher-resolution methods (Alberti et al. 2019; McSwiggen et al. 2019).

While in vitro droplet studies have provided important insights into the biochemical basis of PPIs and biomolecular assemblies, it is increasingly evident that the cellular environment profoundly influences protein behavior. Interpretation is further complicated by in vitro crowding experiments, which often use homogeneous, high-concentration crowders that do not reflect the cell’s true complexity. These approaches ignore important enthalpic interactions and cellular heterogeneity, while common agents like BGB or BSA can alter DNA compaction and protein behavior, making it difficult to relate in vitro results to in vivo mechanisms. Additionally, chemicals like 1,6-hexanediol, often used to dissolve condensates, can disrupt hydrophobic interactions but also cause chromatin damage, cellular toxicity, and impair key signaling pathways. As a result, dissolving condensates with such agents can be misleading, as observed structural changes and cell death may stem from the treatment itself rather than the perturbation of actual biological processes (Ulianov et al. 2021; Shi et al. 2021; Liu et al. 2021; Duster et al. 2021; McSwiggen et al. 2019). Thus, quantitative live-cell imaging and SRM approaches are essential for investigating these assemblies in their physiological context.

Ultimately, it will be essential to assess causality from correlation in chromatin compartmentalization and BA mechanisms of action. Specifically, whether the accumulation of functional components simply reflects “spandrel effects” arising as byproducts of nuclear processes (Solovei and Mirny 2024), or whether they represent functionally significant organizational principles. Bridging these gaps will bring us closer to understanding the true physiological roles of BAs in genome regulation.

## Data Availability

No datasets were generated or analysed during the current study.
